# Evaluation of Psychological Score and Quality of Life in Adults with Allergic Rhinitis and Assessment of Related Risk Factors

**Published:** 2017

**Authors:** Mansoureh Shariat, Zahra Pourpak, Nastaran Sabetkish, Mojtaba Khalesi, Laleh Sharifi, Mostafa Moin

**Affiliations:** 1 Department of Immunology and Allergy, Children Medical Center, Tehran University of Medical Sciences, Tehran, Iran; 2 Immunology, Asthma and Allergy Research Institute, Tehran University of Medical Sciences, Tehran, Iran

**Keywords:** Quality of life, Allergic rhinitis, Olfactory dysfunction, Psychology

## Abstract

**Background:**

To evaluate quality of life (QoL) and psychological score in adults with allergic rhinitis (AR) and to assess the possible risk factors.

**Materials and Methods:**

A total of 110 adult patients with a define diagnosis of AR, who were referred to an outpatient clinic of allergy and immunology, were included in this study. The modified Rhinoconjunctivitis Quality of Life Questionnaire (RQLQ) was applied for the evaluation of QoL in these patients. The psychological score was also assessed, using the Esprint-15 questionnaire.

**Results:**

In a total of 110 patients (68 women, 42 men) with the mean±SEM age of 31.92±1.03 years, nasal congestion was the most common symptom (89.1%). Fifty-one (51%) out of 110 AR patients were found to have olfactory dysfunction (OD; hyposmia, 38%; anosmia, 13%). The relationship between nasal congestion and psychological score was significant (P= 0.005). OD had a significant relationship with QoL, as well as the psychological score (P= 0.001 and P= 0.003, respectively). Furthermore, psychological score had a significant relationship with reduction in QoL and sleep impairment (r, +0.654 and r, +0.591, respectively; P< 0.001). Statistical analysis showed that 54.5% of patients had a high psychological score, which was more common in females, although gender differences were not significant.

**Conclusion:**

Considering the increasing prevalence of AR and the significant relationship of OD and sleep impairment with QoL and psychological score, early diagnosis and treatment of AR may be important in improving QoL.

## INTRODUCTION

Allergic rhinitis (AR), which is the most common form of noninfectious rhinitis, is caused by airborne allergens and is characterized by nasal mucosal inflammation, rhinorrhea, congestion, itching, sneezing, and daily changes in the sense of smell for more than 1 hour as a result of immunoglobuin E (IgE)-mediated nasal inflammation (1). Allergic rhinitis may negatively affect the social life of patients due to decreased social interaction and adverse psychological effects (2).

Additionally, AR may cause significant morbidity, creating major economic burdens, increasing sedative consumption, and disrupting sleep patterns (3, 4). The impact of nasal congestion on quality of sleep has been reported in both children and adults (about 88% in children and 57% in adults) (5). As 43% of AR patients wake up feeling tired, they are exposed to a high risk of depression and anxiety (4).

Several conditions, such as sinonasal diseases, upper respiratory tract infection, trauma, and aging, may cause olfactory dysfunction (OD), which is more frequent in adults with AR, compared to healthy people (6). OD is a key contributor to decreased quality of life (QoL), which reduces the pleasure of eating and drinking and decreases social competence (7). Although the frequency of OD is 19% in population-based studies (8), its frequency is estimated at 20–40% in adults with AR (9). While nasal congestion was thought to be responsible for OD, nasal inflammation has been lately suggested as a contributor (6, 10, 11).

In the current study, we aimed to evaluate QoL and psychological score in adult patients with AR and to assess the possible risk factors.

## MATERIALS AND METHODS

In this cross sectional survey, adult patients, aged 18–60 years, with reversible symptoms of AR, who were referred to the outpatient clinic of allergy and immunology, affiliated to Immunology, Asthma, and Allergy Research Institute (IAARI) of Tehran University of Medical Sciences (Tehran, Iran) were consecutively recruited. Considering a 95% confidential interval and precision of 3%, a total of 110 individuals were enrolled in this study. The study design was approved by the IAARI local ethics committee and research committee, and written informed consents were obtained from all the patients.

In this study, patients with 1 or more AR symptoms, such as itchy nose, rhinorrhea, repeated sneezing, and nasal congestion, were included. The Allergic Rhinitis and its Impact on Asthma (ARIA) guidelines were applied for AR diagnosis (12). In this study, the exclusion criteria were as follows: 1) asthma, active rhinosinusitis, or nasal anatomic structure disorder; 2) history of steroids, antihistamines, thyroid medications, and/or antidepressants for more than 1 month before the study; 3) smoking; and 4) history of nasal surgery.

The demographic data were collected, and comorbidities were also recorded. Individuals with symptoms persisting for less than 4 days or less than 4 consecutive weeks were categorized in the intermittent AR group, while those with AR symptoms for more than 4 days per week or more than 4 consecutive weeks were classified in the persistent AR group. Finally, a total of 110 patients, who met the mentioned criteria, were included in this study.

QoL was evaluated by Rhinoconjunctivitis Quality of Life Questionnaire (RQLQ), developed by Juniper under her permission for the application of the English version (13). Four questions on psychological affectation were also added to the questionnaire from the ESPRINT-15 questionnaire (14). The RQLQ with the integrated questions from the ESPRINT-15 questionnaire was described as the modified RQLQ. It was initially translated into Persian, and 3 immunology and allergy specialists edited the translated questionnaire. Subsequently, back translation was performed by a master of English language with no significant difference from the original questionnaire. The validity and reliability of the questionnaire were evaluated using Cronbach’s alpha in groups of 50 individuals with a confirmed diagnosis of AR. The coefficient alpha was 0.9, suggesting the high internal consistency of the items.

A trained professional physician (a pediatrician and subspecialist in immunology and allergy) completed the modified RQLQ in face-to-face interviews. The mean score of QoL was measured, based on the responses to the modified RQLQ for each individual. A mean score ≥ 3 was considered as high QoL impairment, while a mean score ≤ 3 was considered as low QoL impairment. Moreover, the integrated questions were related to the “management of rhinitis”, “irritability or bad-temperedness because of rhinitis”, “feeling unwell because of rhinitis”, and “general health status with respect to rhinitis”. A mean score of ≥ 3 was considered as a high psychological score, while a mean score of ≤ 3 was considered as a low psychological score.

SPSS for Windows 18.0 (Chicago, USA) was used for data analysis. The results are presented as frequency and mean±standard deviation. Pearson’s Chi square test was used for the analysis of categorical data. *P*-value < 0.05 was considered to be significant.

## RESULTS

A total of 110 consecutive patients, including 68 (61.8%) women and 42 (38.2%) men with the mean±SEM age of 31.92±1.03 years (range, 18–60 years), met the inclusion criteria. The clinical features of the patients are outlined in Table 1. The frequency of sneezing, rhinorrhea, nasal congestion, and watery eyes was 70.9% (n=78), 87.3% (n=96), 89.1% (n=98), and 55.5% (n=61) among patients, respectively. However, no significant relationship was detected between gender and the mentioned symptoms (*P*> 0.05).

**Table 1.** Comparing the prevalence of different clinical features in male and female participants

	**No (%)**		**p value**

	**Male 42 (38.2)**	**Female 68 (61.8)**	
***Clinical symptoms***			
Sneezing	28 (35.9)	50 (64.1)	0.28
Rhinorrhea	36 (37.5)	60 (62.5)	0.45
Nasal congestion	38 (38.8)	60 (61.2)	0.48
Watery eye	22 (36)	39 (64)	0.37
***AR classifications***			
Mild intermittent	2 (28.6)	5 (71.4)	
Severe intermittent	18 (44)	23 (56)	0.7
Mild persistent	6 (30)	14 (70)	
Severe persistent	15 (38.5)	24 (61.5)	
***Comorbidity***			
Sinusitis	23 (36.5)	40 (63.5)	0.21
Asthma	9 (29)	22 (71)	0.15
Conjunctivitis	9 (31)	20 (69)	0.24
Dermatitis	5 (24)	16 (76)	0.1
Urticaria	5 (25)	15 (75)	0.13
Impaired taste	4 (33.3)	8 (66.6)	0.48
Hyposmia	16 (42.1)	22 (57.9)	0.34
Anosmia	5 (38.5)	8 (61.5)	0.6
Reflux	5 (22.8)	17 (77.2)	0.07
***General Health***			
Excellent	1 (50)	1 (50)	
Very good	3 (50)	3 (50)	
Good	6 (33.3)	12 (66.6)	
Normal	21 (39)	33 (61)	0.9
Bad	8 (33.3)	16 (66.6)	
Very bad	3 (50)	3 (50)	
***Psychological affectation***			
Low effect	20 (40)	30 (60)	0.43
High effect	22 (36.7)	38 (63.3)	

Overall, 7 (6.4%) and 41 (37.3%) patients suffered from mild and severe intermittent AR, respectively. Mild and severe persistent AR were reported in 20 (18.2%) and 39 (35.5%) patients, respectively. However, no significant relationship was found between gender and AR grade. Assessment of comorbidities in this subgroup of patients revealed that the prevalence of sinusitis, asthma, conjunctivitis, reflux, dermatitis, and urticaria was 57.3% (n=63), 28.2% (n=31), 26.4% (n=29), 20% (n=22), 19.1% (n=21), and 18.2% (n=20), respectively. Additionally, the frequency of impaired taste, hyposmia, and anosmia was 10.9% (n=12), 34.5% (n=38), and 11.8% (n=13) among patients, respectively, and no significant relationship was found between gender and different comorbidities (*P*> 0.05).

The relationship between QOL score and different variables is summarized in Table 2. Briefly, a significant relationship was detected between OD (hyposmia and anosmia) and reduction in QoL (*P*=0.001 and *P*= 0.02, respectively). However, no significant relationship was found between OD (hyposmia and anosmia) and gender (*P*=0.53 and *P*=0.98, respectively). Although no significant relationship was detected between anosmia and nasal congestion (*P*= 0.69), the statistical analysis revealed a significant relationship between hyposmia and nasal congestion (*P*= 0.008). The relationship between impaired taste and QoL was also significant (*P*= 0.034). However, the statistical analysis showed no significant association between impaired taste and nasal congestion or impaired taste and gender (*P*= 0.19. and *P*= 0.71, respectively).

**Table 2.** Relationship between QoL scores and different variables

	**QoL Score**		**P value**

	**High (mean ≥ 3)**	**Low (mean ≤ 3)**	
**Age**			
18–35 years	31.8%	39%	0.001
36–60 years	22.7%	6.4%	
**Nasal congestion**			
Yes	51.8%	36.3%	0.016
No	2.7%	9.1%	
**Hyposmia**			
YES	26.4%	8.2%	0.001
NO	28.2%	37.3%	
**Anosmia**			
YES	10%	1.8%	0.02
NO	44.5%	43.6%	
**Taste impairment**			
YES	9.1%	1.8%	0.034
NO	45.5%	43.6%	
***AR classifications***			
Mild intermittent	5.4%	3.6%	
Severe intermittent	20.9%	17.3%	0.008
Mild persistent	4.5%	13.6%	
Severe persistent	24.5%	10%	

Table 3 demonstrates the relationship between psychological score and other variables. The majority of patients were severely affected by psychological problems (34.5% in females and 20% in males), while the prevalence of low psychological score was 27.3% and 18.2% in female and male patients, respectively. However, there was no significant association between gender and psychological score (*P*= 0.72). Also, the results revealed a significant relationship between psychological score and QoL, as well as morning symptoms (*P*< 0.0001). The association of psychological score with nasal congestion (*P*= 0.005) and sleep impairment (*P*< 0.0001) was also significant.

**Table 3.** Relationship between psychological score and different variables including gender distribution, QoL, sleep impairment, morning symptoms, and nasal congestion

	**Psychological score**		**P value**

	**High score**	**Low score**	
**Gender**			
Male	20%	18.2%	0.72
Female	34.5%	27.3%	
**QOL**			
High	42.7%	11.8%	<0.001
Low	11.8%	33.6%	
**Sleep impairment**			
YES	38.2%	9.1%	<0.0001
NO	16.4%	36.4%	
**Morning symptoms**			
YES	42.7%	13.6%	<0.0001
NO	11.8%	31.8%	
**Nasal congestion**			
YES	52.7%	36.4%	0.005
NO	1.8%	9.1%	

Based on the findings, the relationship between AR class and OD was significant (*P*= 0.005). In fact, the prevalence of OD was higher in the persistent AR group, compared to the intermittent AR group. The statistical analysis showed that AR class had a significant relationship with QoL (*P*< 0.0001). Similarly, patients with persistent AR were more prone to psychological problems (*P*= 0.001).

## DISCUSSION

The findings of the current study revealed that about half of the participants with AR had OD and high psychological scores (51% and 54.5%, respectively). In addition, the relationships between OD and QOL, OD and psychological score, and psychological score and QOL were found to be significant. Although the frequency of OD in healthy adult males was significantly higher than females (15, 16), no relationship was detected between gender and OD in adults with AR (17–19). Similarly, we did not observe a significant relationship between OD and gender.

The frequency of OD seems to increase with the duration and severity of AR, especially when accompanied by rhinosinusitis (20). Rhee et al. reported a positive correlation between OD and severity of AR (21). The severity of OD was higher in perennial and persistent AR groups in comparison to those with the seasonal disease (9). Nevertheless, contradictory results were reported in a study by Moll and colleagues. In their study, OD was more common in patients with seasonal AR than those with perennial AR in the pollen season (18), while perennial AR was more likely to be accompanied by OD in other seasons (22).

The results of another study revealed that OD occurs in patients with seasonal AR only at the beginning of the pollen season (11). However, Olsson et al. reported no significant relationship between AR and OD (23). Similarly, Stuck and Hummel did not report severe OD in patients with AR (9). The results of the current study showed that AR had a significant relationship with the occurrence of OD, psychological indices, and QoL score. We found that patients with persistent AR were more susceptible to OD, impaired QoL, and deteriorated psychological health.

Previous studies have suggested an association between mood and allergic disorders. Additionally, immune-mediated clinical symptoms are amplified by psychological stress in individuals with allergic disorders. In a study by Bedolla-Barajas et al., over one-third of individuals with rhinitis had anxiety or depression (24), which is compatible with the findings of previous studies, showing a very high frequency of anxiety in patients with AR or vasomotor rhinitis, compared to the controls (25, 26).

In another study, the frequency of anxiety disorders and mood swings was higher in subjects with allergic diseases, compared to patients without allergies (27). Additionally, high frequencies of anxiety and depression were found in patients with OD (28). These findings are in accordance with the results of the current study, indicating a relationship between psychological impairment and other factors, including AR class, QoL score, morning symptoms, sleep impairment, and nasal congestion.

Overall, the risk of depression is greater in women with a history of allergies (25). In a recent study, the predominant incidence of anxiety and depression was highlighted in women in both AR and non-AR groups (24). Similar results were found in the current study in terms of psychological score in females. As the atopic state does not seem to be the only factor involved in anxiety or depression among females, it is essential to conduct further studies to better understand the genesis of psychological disorders in this subgroup of patients.

Interaction of genetic and environmental factors, such as weather conditions and local/cultural aspects, is correlated with allergic sensitization (29). Understanding of the local profile is important in developing environmental control measures, decreasing the incidence of AR, and reducing major consequences, including OD, QoL reduction, and psychological disorders. Although clinical history is an important tool in the diagnosis of AR, meticulous examination of the environmental history is crucial in taking further steps for the enhancement of general health and controlling the symptoms.

In the current study, qualitative ratings were reported by the patients. As a result, quality of the data is limited considering the difficulty in distinguishing nasal patency and olfactory function. In addition, patients were not compared with healthy controls, which is one of the important limitations of the current study. It is important to carry out a population-based study in this subgroup of patients in order to determine the potential risk factors, such as the building age, type of flooring, presence of carpets or rugs, type of materials used in pillows and blankets, presence of furry pets or cockroaches, exposure to second-hand smoking and nonspecific irritants, presence of plants in the household, air-conditioning equipment, and external pollution. Moreover, actions should be taken to eliminate these risk factors and to increase QoL and general health.

## CONCLUSION

In the current study, the relationship between psychological score and nasal congestion, sleep impairment, morning symptoms, and QoL was well described in 110 adult patients with AR. The association of QoL with OD and impaired taste was also documented. These findings highlight the importance of controlling AR symptoms, as they may cause severe QoL and psychological disturbances.
